# Correction: Leptospiral LPS escapes mouse TLR4 internalization and TRIF-associated antimicrobial responses through O antigen and associated lipoproteins

**DOI:** 10.1371/journal.ppat.1009173

**Published:** 2020-12-23

**Authors:** Delphine Bonhomme, Ignacio Santecchia, Frédérique Vernel-Pauillac, Martine Caroff, Pierre Germon, Gerald Murray, Ben Adler, Ivo G. Boneca, Catherine Werts

The legends for the S1–S5 Figs are missing. Please see the full legends here.

**S1 Fig. Leptospiral LPS does not trigger TLR4 internalization**.

A) Representative images of confocal IF analyses of BMDMs either WT or TLR4 KO non stimulated and stained for mouse-TLR4 (mTLR4), glycoproteins (Weat Germ Agglutinin, WGA) and nuclei (DAPI). B) Scheme of the quantification method for mTLR4 fluorescence profiles on cross section of RAW264.7 cells either non-stimulated (NS) or stimulated 1h with 100 ng/mL of LPS of E. coli. C) Silver staining analyses of LPS of various serovars of *L*. *interrogans* (serovar Icterohaemorrhagiae strain Verdun, serovar Copenhageni strain Fiocruz L1-130, serovar Manilae strain Manilae L495) after SDS-PAGE with 1µg of LPS/well. Positions of standard molecular mass markers are shown on the left. D) Complete gating strategy for flow cytometry analyses of RAW264.7 cells: elimination of cell debris (first panel), selection of single cells (second and third panels), selection of live cells (fourth panel) and determination of basal levels of isotype and surface mTLR4 staining on non-stimulated cells (fifth panel). E) Flow cytometry analysis of surface mTLR4 on RAW264.7 cells upon either non-simulated (NS) (grey), stimulated with 1µg/mL of LPS of *E*. *coli* (blue) or *L*. *interrogans* (green) at 1h, 4h and 24h post stimulation. Data shown are MFI and each dot corresponds to one well (10 000–30 000 events). F) Flow cytometry analysis of surface mTLR4 (or isotype) on RAW264.7 cells upon either non-simulated (grey), stimulated with either 100ng/mL or 1µg/mL of LPS of *E*. *coli* (blue) or *L*. *interrogans* (green) at 1h. Data shown are MFI and each dot corresponds to one well (10 000–30 000 events).

**S2 Fig. Leptospiral LPS avoids TRIF-dependent responses but activates MyD88**.

A) Production of KC by WT, TLR2 KO and TLR4 KO BMDMs after 24h stimulation with 1 µg/mL of LPS of *L*. *interrogans* (green), LPS of *E*. *coli* (blue) or 100 ng/mL of Pam3Cys (grey). B) Productions of KC and RANTES by WT and TLR2/TLR4 DKO BMDMs after 24h stimulation with 1 µg/mL of LPS of *L*. *interrogans* (green), LPS of *E*. *coli* (blue) or 1 µg/mL of Poly(I:C) (grey). C) Productions of KC, RANTES and NO by WT and TrifLps2 BMDMs after 24h stimulation with 1 µg/mL of LPS of *E*. *coli* (blue) or 100 ng/mL of Pam3Cys (grey). D) mRNA levels of IFNβ in WT and TrifLps2 BMDMs after 24h stimulation and cytokine production after 8h stimulation with 1 µg/mL of LPS of *L*. *interrogans* (green) or LPS of *E*. *coli* (blue). E) Productions of KC and NO by WT and TrifLps2 BMDMs after 24h stimulation with lower doses (100 ng/mL) of LPS of *L*. *interrogans* (green) or LPS of *E*. *coli* (blue). Data are represented as mean (+/- SD) of n = 3/4 technical replicates and are representative of at least 3 independent experiments. Statistical analyses were performed using the non-parametric Mann-Whitney test.

**S3 Fig. Shorter leptospiral LPS induces more TLR4-TRIF responses**.

A) Silver staining analyses of LPS of *L*. *interrogans* serovar Icterohaemorrhagiae strain Verdun and *L*. *biflexa* serovar Patoc strain Patoc after SDS-PAGE with the equivalent of 1 µg of LPS/well. Positions of standard molecular mass markers are shown on the right. B) Human embryonic kidney cells (HEK293T) reporter system for mouse-TLR4 (left axis) and human-TLR2 (right axis) activities of 1 µg/mL of LPS of *L*. *interrogans* strain Verdun (upper panel, dark green) or *L*. *biflexa* strain Patoc (lower panel, light green), with the corresponding controls: 100 ng/mL of LPS of *E*. *coli* for mTLR4 activity and 100 ng/mL of Pam3Cys for hTLR2 activity. C) Production of RANTES by WT, TLR2 KO and TLR4 KO BMDMs after 24h stimulation with 1 µg/mL of LPS of *L*. *interrogans* strain Verdun (dark green), LPS of *L*. *biflexa* strain Patoc (light green), LPS of *E*. *coli* (blue) or 100 ng/mL of Pam3Cys (grey). D) Production of RANTES and NO by RAW264.7 cells after 24h stimulation with 1 µg/mL of LPS of *L*. *interrogans* strain Verdun (dark green) or *L*. *biflexa* strain Patoc (light green). Data are represented as mean (+/- SD) of n = 3/4 technical replicates and are representative of at least 3 independent experiments.

**S4 Fig. Soluble CD14 participates in the enhanced signaling of the shorter leptospiral LPS**.

Production of RANTES by RAW264.7 cells after 24h stimulation in various serum conditions, and stimulated with 1 µg/mL of LPS of L. interrogans L495 (dark green), shorter LPS of L. interrogans M895 (light green), or of LPS E. coli (blue). Data are represented as mean (+/- SD) of n = 3/4 technical replicates and are representative of at least 3 independent experiments.

**S5 Fig. Copurifying lipoproteins contribute to the escape of TLR4 internalization**.

A) Human embryonic kidney cells (HEK293T) reporter system for mouse-TLR4 (left axis) and human-TLR2 (right axis) activities of 1 µg/mL of the LPS of L. interrogans (dark green) after either proteinase K or B) lipase treatments (light green), with the corresponding controls: 100 ng/mL of LPS of E. coli for mTLR4 activity and 100 ng/mL of Pam3Cys for hTLR2 activity. Effect of proteinase K or lipase alone on the cells was controlled (mock). C) Production of RANTES by RAW264.7 cells stimulated for 24h with 1µg/mL of LPS of L. interrogans (dark green) after either proteinase K or D) lipase treatment (light green) and LPS of E. coli (blue). Effect of proteinase K or lipase alone on the cells was controlled (mock). E) Silver staining analyses after SDS-PAGE of LPS (1µg of LPS/well) treated with increasing doses of proteinase K. Positions of standard molecular mass markers are shown on the left. F) HEK293T reporter system without transfection allowing to analyze TLR5 activation (that is constitutively expressed to low levels in HEK293T) upon stimulation with 100 ng/mL flagellin (red) digested or not with proteinase K. G) Western Blot after SDS-PAGE with the equivalent of 5 µg of LPS/well showing LipL32 contamination in the original and repurified LPS preparations. Positions of standard molecular mass markers are shown on the right. H) HEK293T reporter system for mTLR4 (left axis) and mTLR2 (right axis) activities of 1 µg/mL of the LPS of L. interrogans serovar Manilae strain L495 WT or LPS of the strain M933 ΔLipL32 with the corresponding controls: 100 ng/mL of LPS of E. coli for mTLR4 activity and 100 ng/mL of Pam3Cys for mTLR2 activity. I) Production of RANTES by RAW264.7 cells stimulated 24h with 1 µg/mL of LPS of the WT strain (dark green), LPS of the M933 ΔLipL32 strain (light green) or LPS of E. coli (blue). Data are represented as mean (+/- SD) of n = 3/4 technical replicates and are representative of at least 3 independent experiments.
