# Supplementary material for: Leptospiral LPS escapes mouse TLR4 internalization and TRIF‑associated antimicrobial responses through O antigen and associated lipoproteins
Source: PLoS Pathog. 2020 Aug 13;16(8):e1008639. doi: 10.1371/journal.ppat.1008639 (PMC7447051; doi:10.1371/journal.ppat.1008639)
Supplement: S1 Table — (DOCX) [file ppat.1008639.s001.docx]

**S1 Table. List of primers for RT-qPCR experiments**

| mRNA | Accession  number | Forward  (nt position) | Reverse  (nt position) | Probe  (nt position) | Probe  type |
| --- | --- | --- | --- | --- | --- |
| **HPRT** | 013556 | 636-656 | 744-719 | 659-687 | FAM TAMRA |
| **RANTES** | 013653 | 242-263 | 312-286 | 265-284 | FAM TAMRA |
| **iNOS** | 010927 | 2176-2196 | 2270-2250 | 2198-2220 | FAM TAMRA |
| **HPRT** | Pre-designed (TaqMan Gene Expression Assays) | | | | FAM NQMGB |
| **IFNβ** | Pre-designed (TaqMan Gene Expression Assays) | | | | FAM NQMGB |
