# Supplementary figures and images for: Leptospiral LPS escapes mouse TLR4 internalization and TRIF‑associated antimicrobial responses through O antigen and associated lipoproteins
Source: PLoS Pathog. 2020 Aug 13;16(8):e1008639. doi: 10.1371/journal.ppat.1008639 (PMC7447051; doi:10.1371/journal.ppat.1008639)

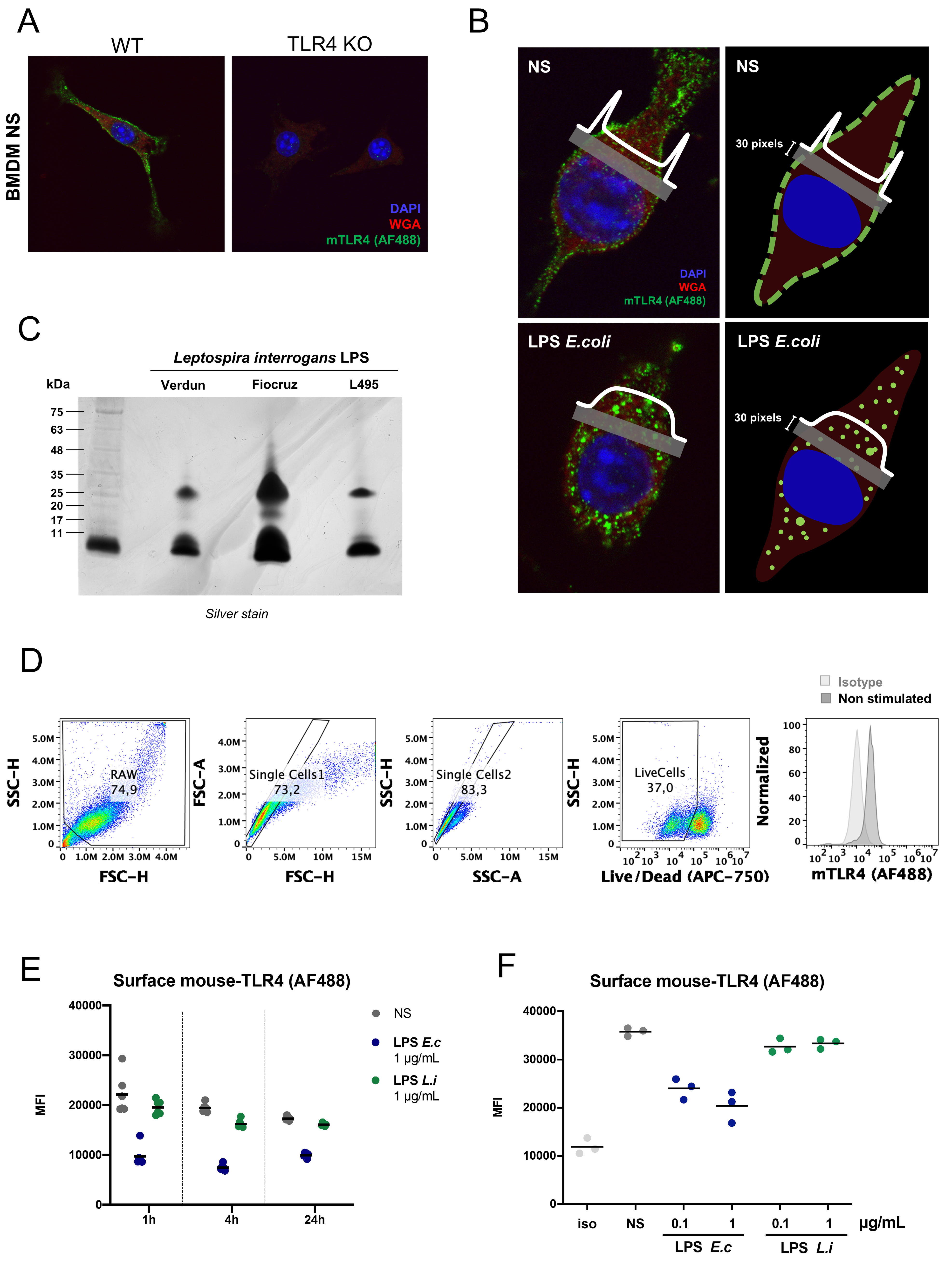

Supplement: S1 Fig — (TIF) [file ppat.1008639.s002.tif]

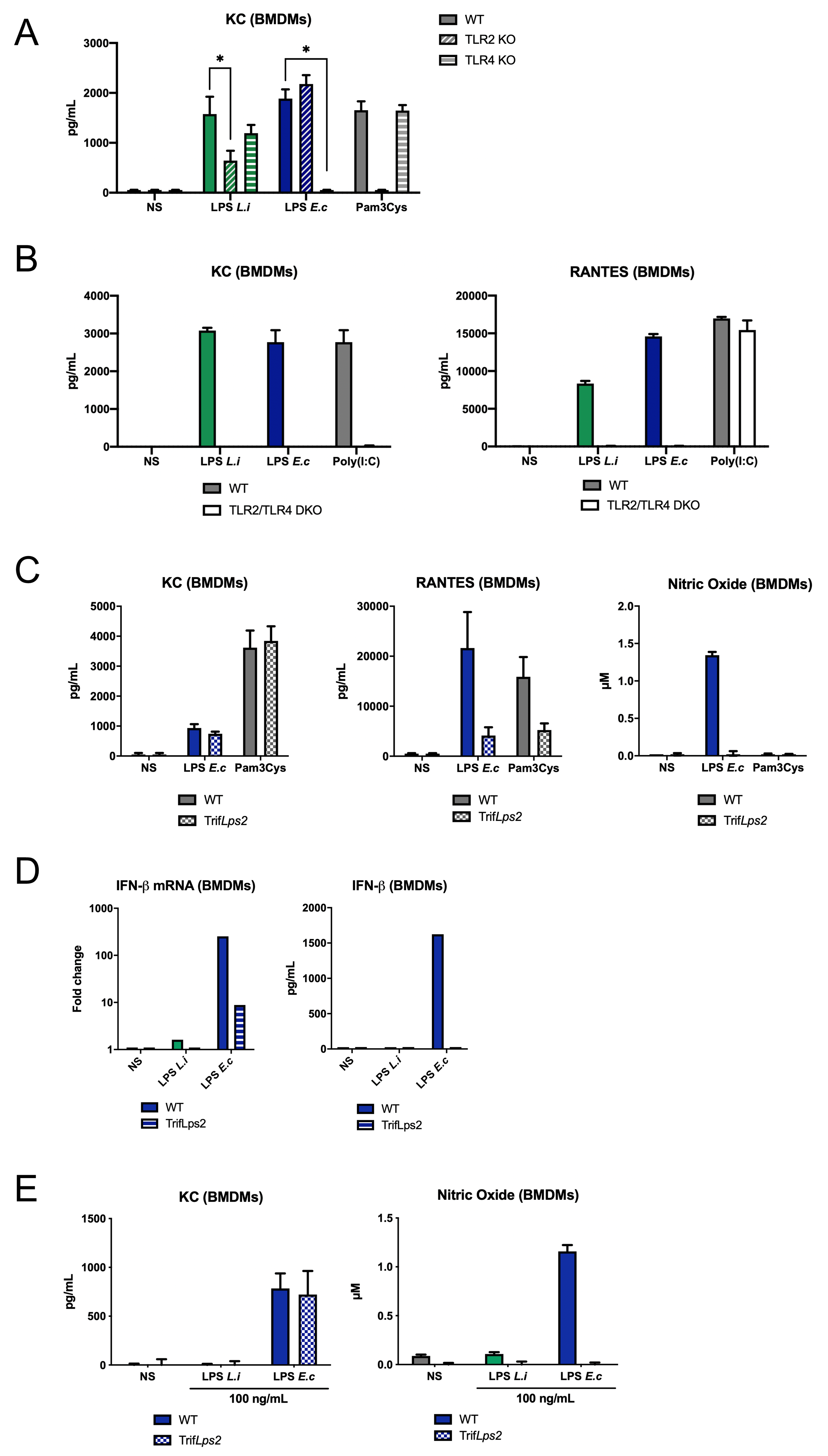

Supplement: S2 Fig — (TIF) [file ppat.1008639.s003.tif]

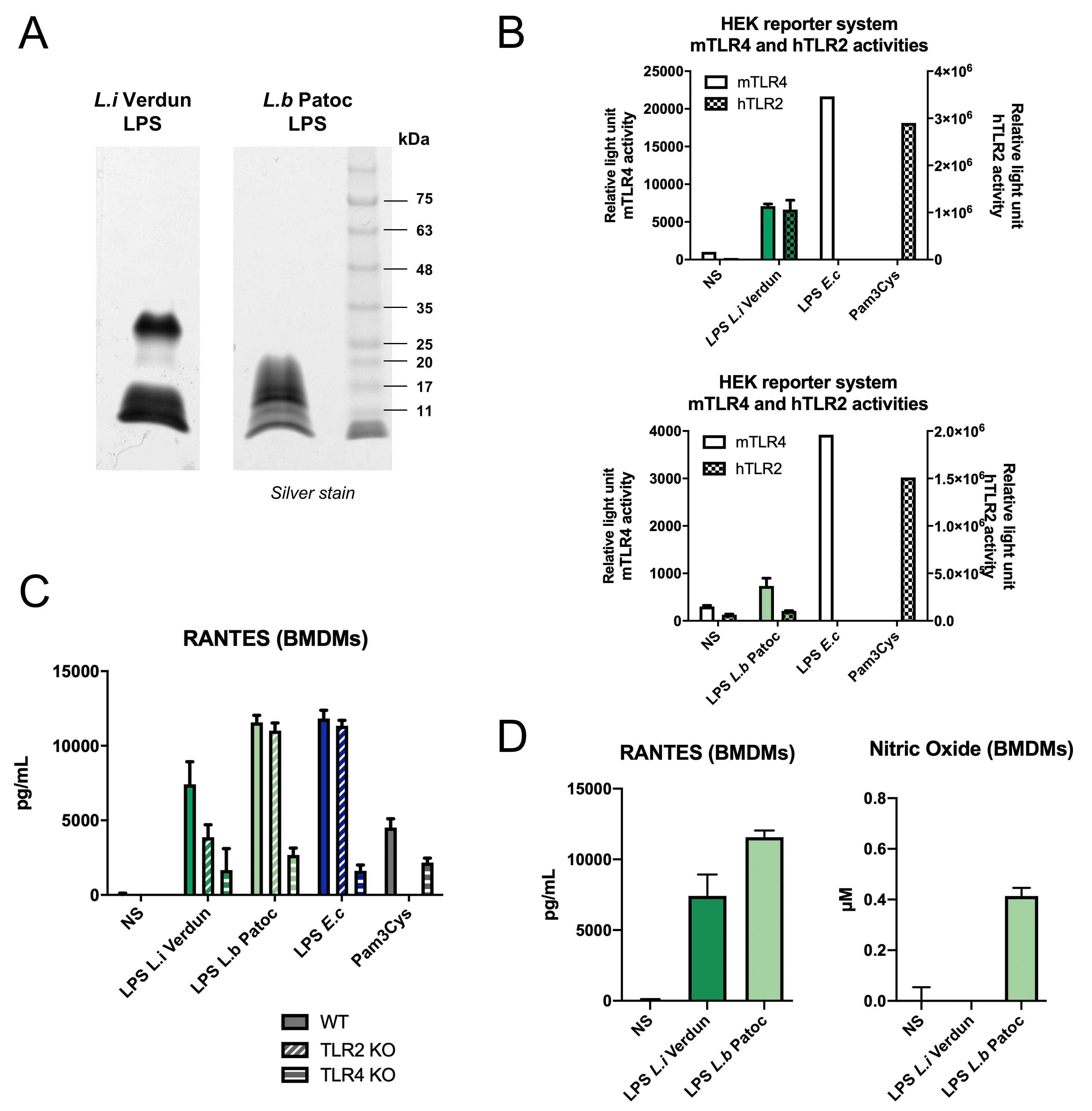

Supplement: S3 Fig — (TIF) [file ppat.1008639.s004.tif]

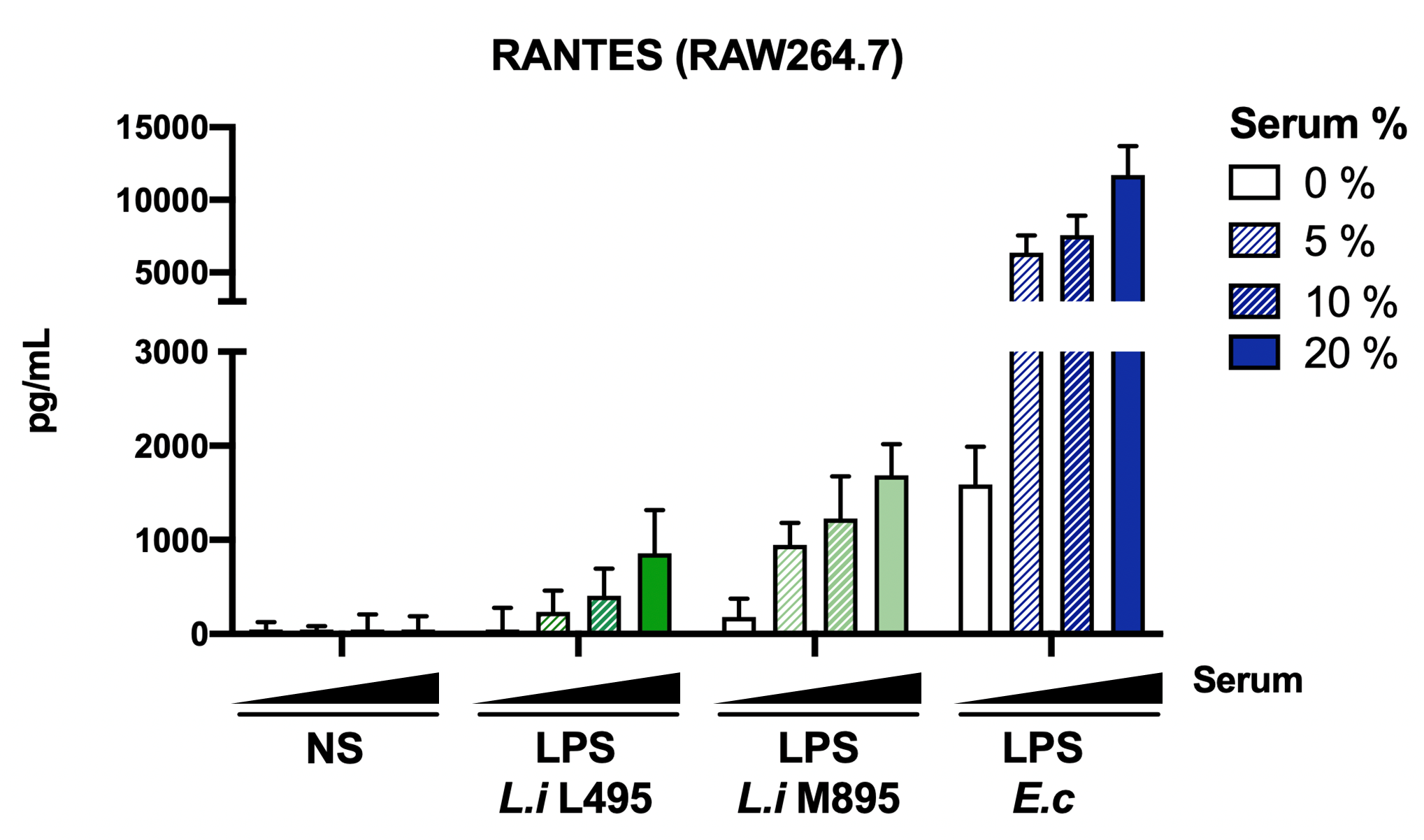

Supplement: S4 Fig — (TIF) [file ppat.1008639.s005.tif]

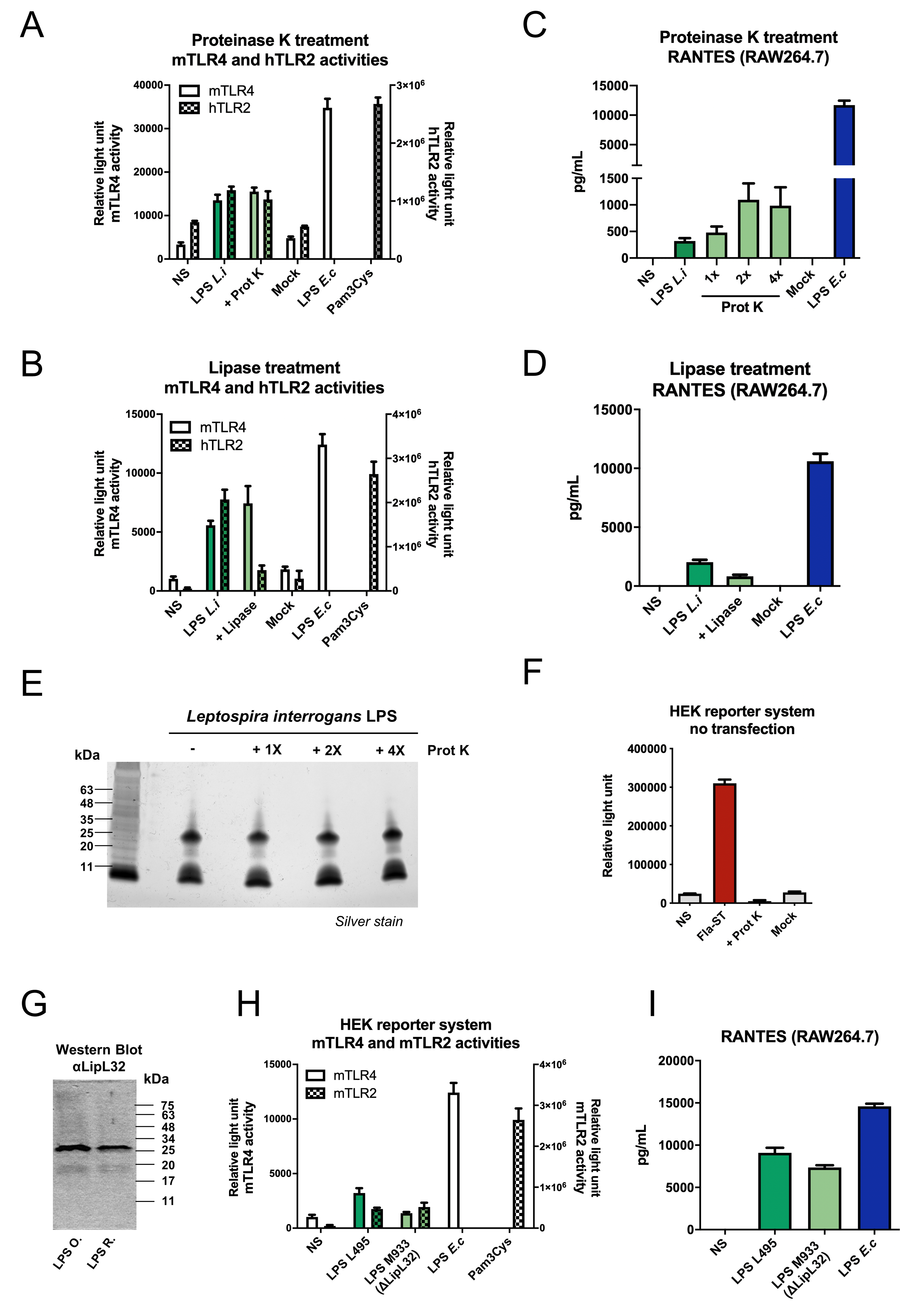

Supplement: S5 Fig — (TIF) [file ppat.1008639.s006.tif]
