# Supplementary material for: Leptospiral LPS escapes mouse TLR4 internalization and TRIF‑associated antimicrobial responses through O antigen and associated lipoproteins
Source: PLoS Pathog. 2020 Aug 13;16(8):e1008639. doi: 10.1371/journal.ppat.1008639 (PMC7447051; doi:10.1371/journal.ppat.1008639)
Supplement: Attachment — Submitted filename: AnswersToReviewers-TLR4-02072020.docx [file ppat.1008639.s007.docx]

**Dear reviewers,**

**First of all, we would like to thank the reviewers for all positive and constructive comments regarding our manuscript. We answered most of the questions, and we provide several additional experiments, as suggested.**

**The main addition to the figures was the IF analysis of the TLR4 internalization induced by the M895 strain that was added in Figure 6. Also, in order to make our message clearer, we also added a schematic summary of the leptospiral LPS recognition in human and mouse cells as Figure 7.**

**Some other modifications were also done: the microscopy images contrast and brightness were enhanced as requested and LPS of *E. coli* was added as a positive control on all figures (Figures 1A, 3C, 5D, and 6A). The quality of the WB for CD14-LPS binding assay was improved (Figure 4C). Also, the various TRIF dependent readouts (NO, RANTES and IFNβ) were more consistently shown in all the figures (Figures 2A, 2B,2C, 3D,3E, 3F, 4A, 4B and 6C). Several controls were added: LPS in signaling in TLR2/TLR4 DKO and IFNβ ELISA (Figure S2B and Figure S2D) and some missing statistical analyses were performed (Figure 2B, 2C, 3E, 3F, and S2A). Finally, we added the previously not shown WB of the contaminating LipL32 in the original and repurified LPS preparations (Figure S5G).**

**We accordingly revised the manuscript. First, the Results and Legends sections were modified to correspond to the previously described figures modifications. Also, and as suggested, the difference between human and mouse in the study of the leptospiral LPS signaling was more clearly described in the Abstract, Summary and Discussion sections. Finally, the Discussion was also modified in order to better explain our hypothesis that the partial escape of the leptospiral LPS recognition in mice could lead to the chronicity of the infection.**

**Please find all our detailed answers in color in the text.**

**Part I - Summary**
Please use this section to discuss strengths/weaknesses of study, novelty/significance, general execution and scholarship.


Reviewer #1: The host response to bacterial lipopolysaccharide (LPS) is mediated via signaling through the pattern-recognition receptor Toll-like receptor 4 (TLR4) via two pathways: a MyD88-dependent route upon recognition of the antigen by TLR4 at the cell surface, and a TRIF-dependent route upon subsequent internalization of LPS-bound TLR4. The authors describe previous work characterizing the unique manner in which the LPS of Leptospira interrogans interacts with the host response, including that although this LPS signals through murine TLR4, it is less endotoxic than the LPS of certain other microbes. In addition, certain lipoproteins that co-purify with leptospiral LPS have been shown to aid in evasion of the early host response. In this report, the authors provide a methodical and thorough investigation of these aspects of the TLR4-mediated host response to LPS of L. interrogans.

Here, the authors show that, unlike the LPS of Escherichia coli, the LPS of a pathological L. interrogans strain did not induce internalization of TLR4. This lack of TLR4 internalization was also observed using the LPS of other L. interrogans serovars, different concentrations of LPS, and different durations of incubation with host cells. High concentrations of leptospiral LPS were shown to less effectively stimulate TRIF-dependent (TLR4-internalized) pathways than LPS of E. coli, leading to less production of nitric oxide (NO) and RANTES, while still stimulating MyD88-dependent pathways. The authors then showed that strains with shorter LPS allowed for more effective internalization of TLR4 and greater production of NO and RANTES than did pathogenic strains with full-length LPS, leading to the hypothesis that possession of full-length LPS allowed for the evasion of TLR4 internalization and the ensuing activation of TRIF-dependent responses. Soluble CD14, a key component of TLR4 signaling, was shown to significantly aid in the production of a TRIF-dependent product in response to the shorter LPS but not to the full-length LPS; the authors hypothesized, after performing binding assays, that the latter effect could be due to a lower affinity between full-length LPS and sCD14. Additionally, leptospiral LPS that was re-purified, with the effect of significantly removing associated lipoproteins, led to increased internalization of TLR4 and increased transcription/production of various TRIF-dependent immune factors. Digestion of LPS with proteinase K led to increased RANTES production, indicating that the protein components of lipoproteins associated with LPS aid in the escape from TRIF signaling. Finally, the findings were supported with a study using whole L. interrograns microbes.

Collectively, these novel findings describe important mechanisms by which L. interrograns can effectively evade critical factors of the host response. The authors’ findings were supported using various experimental methods and were bolstered by robust primary and supplementary data. This was an exceptionally well written and logical manuscript that is suitable for publication with some minor modifications.

Reviewer #2: This is a well-conceived paper by a group of experienced investigators that have a long track record studying the biochemical characteristics of Leptospiral LPS and the host immune response to Lepto LPS in mouse and human cells. They hypothesize that Leptospira LPS, known as a virulence factor, plays a major role in the innate immune evasion of the leptospires, thereby contributing to their stealthy-ness and chronicity in mice. It is a very important study as they provide novel mechanistic data showing that Leptospira LPS escapes the TRIF arm of the TLR4 response to evade RANTES and NO antimicrobial responses. The paper is generally well written and the figures, captions and legends help the reader quickly follow the main objective of the study. One weakness throughout is the lack of clarity between distinction of assessments between mouse and human TLR4. Another is lack of consistency in defining what leads to immune evasion that leads to tolerance and resistance (decreased susceptibility) to infection and evasion from one pro-inflammatory pathway (but not the other) that would lead to increased susceptibility to infection.

Reviewer #3: The manuscript by Bonhomme and colleagues examines cell signaling downstream of the binding of leptospiral LPS to Toll-like receptors with the aim to understand its low endotoxicity. The authors show that leptospiral LPS does not trigger internalization of TLR4, which is potentially linked to its reduced ability to trigger TRIF-dependent responses while MyD88-dependent responses remain intact. Moreover, the group shows that contaminating lipoproteins contribute to the TLR2 response of the LPS but also appear to impair TLR4 internalization and TRIF-dependent responses.

Overall, these are nice findings but there are a few issues to address.

**Part II – Major Issues: Key Experiments Required for Acceptance**
Please use this section to detail the key new experiments or modifications of existing experiments that should be absolutely required to validate study conclusions.

Generally, there should be no more than 3 such required experiments or major modifications for a "Major Revision" recommendation. If more than 3 experiments are necessary to validate the study conclusions, then you are encouraged to recommend "Reject".


Reviewer #1: 1. At the risk of adding to the abundant data presented here, it was noted that the TRIF-dependent products examined were not presented or described regularly throughout the main figures. It appears that levels of NO were consistently shown, but data on RANTES and, moreso, IL-1beta were not. And, no data on production of IL-1beta protein were shown. A lack of this information does not necessarily negate the main conclusions of this study, but it seems that inclusion of such additional data would further strengthen their conclusions. Would the authors please comment on why these products were not more consistently shown?

**The various TRIF dependent readouts were not all presented in all the figures of the original manuscript because we focused mainly on NO and RANTES for technical reasons. However, we agree that showing these readouts more consistently contributes to the strength of the message. Therefore, we repeated some experiments and completed the figures in the revised manuscript in order to show more consistently the different cytokines and mRNA levels (Figure 3E and 3F, Figure 4A et 4B and Figure 6C). Furthermore, regarding the analyses of IFNβ, we have observed that the mRNA levels are representative of the cytokine production, and provided this control as new Figure S2D.**


2. In Figure 6, the effect of the shorter M895 on NO production was shown, but what about the effect of this microbe on internalization of TLR4?

**Indeed, this information was missing. We performed the experiment demonstrating that the M895 mutant does induce the internalization of the mTLR4, consistent with its higher NO production that that WT strain, and added such results in the revised manuscript (Figure 6).**

Reviewer #2: Since there are comparisons between human and mouse TLR4 and TLR2 the authors need to clearly identify throughout the paper what is mouse and what is human. For example, the Abstract and Summary both start with emphasis on human and then end up explaining data seen in mouse. In Discussion it is also important to lead in the first paragraph with the clarification that the data discussed is mouse data (and comparisons and differences w human need to be highlighted throughout).

**Thank you for commenting on our misleading sentences. We tried to be more careful and revised the manuscript in order to make clearer this difference between human and mouse. Modifications were made as requested in the Abstract, Summary and Discussion.**

Reviewer #3: Figure 1: The intensity of the IF stain needs to be increased; it was difficult to see in my copy. Also, please indicated what WGA is staining?

**These modifications were done. Contrast and brightness were both increased by 10% homogeneously on all figures, and such modification was noted in the Materials and Methods section. Also, we indicated that WGA (Wheat Germ Agglutinin) targets all glycoproteins in permeabilized cells, and it was also added in the Materials and Methods section.**

While KC induction is fully dependent on MyD88, it’s not clear how much of that signal is TLR4 vs TLR2 dependent. In Figure S2A, the stats are not included for the KC values in the TLR2 and TLR4KO. Did the authors test TLR2/4 double KOs to ensure all of the signal is going through these receptors? `

**The production of KC induced by the leptospiral LPS is indeed dependent on TLR2 and TLR4, although it is only significantly lost in the TLR2 KO BMDMs (statistical analyses were added on Figure S2A as requested). Furthermore, the test of LPS signaling in DKO (TLR2/TLR4) BMDMs was performed and confirmed, as already shown (Nahori et al., 2005) that the LPS of leptospires only signal *via* these two receptors. This experiment was added as Figure S2B.**

Ensure to provide stat tests for mRNA levels in 1B.

**As requested, all the mRNA levels experiments were repeated in conditions allowing to perform statistical analyses, and confirmed in Figure 1B and 1C that the mRNA levels of iNOS, RANTES and IFNβ are significantly different between LPS of *L. interrogans* and LPS of *E. coli*.**

The authors also show at low concentrations, leptospiral LPS does not activate either pathway – how does this concentration of LPS relate to an infectious dose?

**As added in the revised manuscript, we estimate, according to our yields of purification, that a MOI comprised between 10 to 50 could correspond to 1µg/mL of LPS, although we indicated clearly in the results section (corresponding to Figure 6) that such comparison must be interpreted with caution.**

Also, would it be informative to see in vivo with leptospiral LPS injection in mice that it does not induce TRIF-dependent responses? This might hint at importance during in vivo infection.

**We agree that such experiment would indeed have been extremely interesting. However, we unfortunately do not have ethical authorization to perform LPS injection in mice. We hence apologize but were not able to provide this experiment in the revised manuscript. Moreover, since some of the responses induced by the short leptospiral LPS (like IFNβ) are similar to the ones induced by LPS of *E. coli*, it suggests that injection of the M895 LPS could have been more harmful for the mice than the WT leptospiral LPS, and most probably we would have not obtained this authorization.**

Figure 3C – Again here the IF was not very easy to see. Also, should include an E coli LPS to compare with the M895 strain.

**All these modifications were done.**

Figure 3D – Would also be helpful to have comparison with KC; also mRNA levels would add to data.

**These experiments were performed in RAW264.7 cells that do not produce KC. However, we could analyze mRNA levels as suggested and we added RANTES, iNOS and IFNβ mRNA levels as Figure 3E and 3F.**

**Figure 4: Western blot for CD14 needs to be improved.**

**We apologize for the poor quality of the WB provided in the original manuscript which was performed just before the lockdown. We repeated the experiment and we provided a new figure in the revised version.**

Figure 5D: Again the signal here is difficult to see. G) Stats required.

**Statistical analyses were not performed on these experiments because the qRT-PCR (although repeated 3 times) were performed in duplicates. We apologize for not anticipating such issue.**

Figure 6A: For the IF, would be good to have L495 as a comparison.

**We agree and added the comparison between the WT L495 and mutant M895 strain in both immunofluorescence analyses and cytokine production.**

**Part III – Minor Issues: Editorial and Data Presentation Modifications**
Please use this section for editorial suggestions as well as relatively minor modifications of existing data that would enhance clarity.


Reviewer #1: 1. Line 141: Figure 1SA doesn’t seem show the data described in this sentence, which describes stimulated cells.
2. Line 147: “cells” needed after RAW264.7.
3. Line 173: Were these cells RAW264.7 cells? The caption for Figure 2B states BMDMs.
4. Line 205: What is meant by “comparable activity” here? The values are different, although the comparison to NS conditions is the same.
5. Line 284: Perhaps “from”, instead of “to”.
6. Line 417: Perhaps “another”, instead of “other”.
7. Line 505: Perhaps “macrophage”, instead of “macrophages”.
8. Lines 835, 837, 893, 907: It is unclear what is meant by “panel” here.
9. Line 839: Missing an open parenthesis.
10. Line 877: “Blot”, rather than “Bot”.
11. Line 963: L495 LPS isn’t mentioned here.
12. Lines 970-974: The description of the dark/light green bars was somewhat confusing. Should the lipase bars in Figures S5B and S5D be light green?
13. Figure S5G: Should “M933” be included in the data label for the LipL32 mutant, as stated in Line 981?
14. Lines 983-984: Wording of “green bars” (plural) to describe each individual group was somewhat confusing.

**We thank you for the very careful corrections you provided on our manuscript and took in consideration all the suggestions and corrections on the revised manuscript.
12. You are right, the color code was not correct in the figures of the original manuscript and was modified accordingly all over the figures.
13. The “M933” label was added on the figure as suggested.**

Reviewer #2: Minor:
Abstract
Line 30-31: needs clarification.

Introduction
In general, this section is very long. Some of this information may be better used in discussion to explain the results observed?

**As suggested, and in order to shorten the Introduction section, the paragraph about the LPS Binding Protein was moved to the Discussion section**.

Line 61: I think a more accurate term in this context is enzootic (rather than zoonotic)
Line 72 and 109: consider unconventional instead of peculiar
Line 91: delete interferons (because IFN is a cytokine)
Line 105: consider deleting stricto sensu because it translates into essential
Line 122: consider replacing mystery with unknown

Results
Line 135: after membrane TLR4 add (mTLR4?), it needs to be clarified if mTLR4 stands for membrane or mouse? given that in Fig S3B mTLR4 stands for mouse.

**We apologize for the lack of clarity of our original manuscript and figures. Indeed, mTLR4 stands for mouse-TLR4. We made things clearer in the revised version by specifying that at the beginning of the text and by talking about surface TLR4 instead of membrane TLR4.**

Line 163 (Fig 2): why only 24h? was the analysis not done at 1h also?

**All the cytokines and mRNA analyses were performed at 24h post stimulation (except for IFNβ ELISA that was done at 8h). Indeed, although the internalization of the receptor is visible at 1h, the production of cytokines and mRNA at detectable levels requires more time. In our study, the time of 24h post stimulation was chosen because it is the best time point to check for NO production in the supernatant of stimulated macrophages.**

Lines 186-188: this setup is a bit confusing, it needs clarification.
Fig 3B: adding the E.coli control here helps visualize the differences
Fig 3C: There not much difference between NS L495 and M895 mTLR4 staining in this figure. This may be easier to visualize against the Ecoli control.

**The control of *E. coli* LPS was added as a positive control in both IF and cytokines production figures as suggested.**

Line 204-206: This comparison needs to be done on the same graph with the same axis. It looks like mTLR4 in LPS Patoc is 5-6X lower than LPS Verdun and hTLR2 is 8X lower?

**Unfortunately, these graphs represent two independent experiments and we therefore cannot plot them on the same graph. However, and as better described in the revised manuscript, the comparison of the activities must be done with the internal controls (LPS of *E. coli* and Pam3cys).**

Line 246: add Fig 5c after treatment (for the silver stain fig)
Fig 5D and 5E: it helps to visualize this against the E. coli control. This is mouse and human. It needs to be clarified

**Again, LPS of E. coli was added as a positive control for IF here.
Furthermore, as described in the reviewed version, no species specificity was observed between hTLR2 and mTLR2, and we never saw differences between the two receptors, hence allowing us to work indifferently with either one. This justifies why we presented mTLR4 and hTLR2 activities in Figure 5A.**

Line 254: replace more produced with increased
Fig 6B: it helps to visualize this against the E. coli control
Line 284: consider replacing participates with contributes

Discussion:
The authors need to better differentiate the use of the term “escape immunity” that lead to tolerance or resistance in mouse from “escape” that leads to susceptibility and severe disease, this becomes quite confusing to the reader. Maybe use escape immunity + stealth of the pathogen, but not escape+virulence of the pathogen.

**We thank you for pointing out the lack of clarity in our discussion.**

**First, we tried to make a clearer distinction between:
The total lack of TLR4 recognition in sensitive humans, that leads to full leptospiral escape from immunity and potentially to fatal leptospirosis.
The partial lack of TLR4 recognition in resistant mice, that (although it prevents from acute leptospirosis) could account for a partial escape of the leptospires and contribute to chronicity of the infection.
A concluding schematic representation of the species difference was added as Figure 7 of the revised version.**

**Then, we also tried to remove the term “virulence” that is not appropriate for our study in the resistant mouse model, and replace it by “attenuation”. We therefore explain that the M895 strain is attenuated in the sensitive hamster model (in which it has lost its virulence) but also in the resistant mouse model (in which it no longer colonizes the kidneys).**

Discuss the Patoc data.

**We added a brief discussion of the Patoc data in the paragraph regarding the other strain with an LPS devoid of TLR2 activity that leads to self-resolving leptospirosis.**

Line 415: Clarify that this is mouse in the beginning of the sentence.

Methods
HEK293T: clarify that these are human cells.
Where’s your mouse RAW264 macrophages?

**We modified the title and legends of the HEK experiments, to make clearer that this was a human cells reporter system, in which we expressed the mouse TLR4 or mouse or human TLR2 unlike the other experiments performed in mouse macrophages.**

Reviewer #3: see above
